# Performance and ease of use of a molecular point-of-care test for influenza A/B and RSV in patients presenting to primary care

**DOI:** 10.1007/s10096-020-03860-5

**Published:** 2020-03-14

**Authors:** Jan Y Verbakel, Veerle Matheeussen, Katherine Loens, Mandy Kuijstermans, Herman Goossens, Margareta Ieven, Christopher C Butler

**Affiliations:** 1 NIHR Community Healthcare MIC, Nuffield Department of Primary Care Health Sciences, University of Oxford, Woodstock Road, Oxford, Oxfordshire OX26GG UK; 2 Department of Public Health and Primary Care, KU Leuven, Kapucijnenvoer 33, 3000 Leuven, Belgium; 3 Nuffield Department of Primary Care Health Sciences, University of Oxford, Woodstock Road, Oxford, Oxfordshire OX26GG UK; 4 Laboratory of Clinical Microbiology, Antwerp University Hospital, Edegem, Belgium; 5 Laboratory of Medical Microbiology, Vaccine & Infectious Disease Institute (VAXINFECTIO), University of Antwerp, Antwerp, Belgium

**Keywords:** Influenza, Point-of-care, Primary care, Ease-of-use, PCR

## Abstract

Annual influenza epidemics cause substantial morbidity and mortality, and the majority of patients with influenza-like illness present to primary care. Point-of-care influenza tests could support treatment decisions. It is critical to establish analytic performance of these platforms in real-life patient samples before uptake can be considered. We aimed to assess the analytical performance and ease of use of the cobas® Liat® PCR POCT in detecting influenza A/B and RSV in samples collected from patients with influenza-like illness in primary care. Sensitivity and specificity of the cobas® Liat® POCT are calculated in comparison with a commercial laboratory-based PCR test (Fast-Track Respiratory Pathogens 21 Plus kit (Fast-Track Diagnostics)). Samples with discordant results were analysed additionally by the RespiFinder 2Smart (PathoFinder) using an Extended Gold Standard (EGS). Acceptability was scored on a five-point Likert scale as well as a failure mode analysis of the cobas® Liat® POCT was performed. Nasal and oropharyngeal swabs were obtained from 140 children and nasopharyngeal swabs from 604 adults (744 patients). The cobas® Liat® POCT had a sensitivity and specificity of 100% (95% CI 99–100%) and 98.1% (95%CI 96.3–99%) for influenza A, 100% (95% CI 97.7–100%) and 99.7% (95%CI 98.7–99.9%) for influenza B and 100% (95% CI 87.1–100%) and 99.4% (95%CI 98.6–99.8%) for RSV, respectively. According to trained lab technicians, the cobas® Liat® POCT was considered easy-to-use, with a fast turn-around-time. Cobas® Liat® POCT is a promising decentralised test platform for influenza A/B and RSV in primary care as it provides fairly rapid results with excellent analytic performance. Point-of-care influenza tests could support treatment decisions in primary care. Cobas® Liat® POCT is a promising decentralised test platform for influenza A/B and RSV in primary care as it provides fairly rapid results with excellent analytic performance.

**Electronic supplementary material:**

The online version of this article (10.1007/s10096-020-03860-5) contains supplementary material, which is available to authorized users.

## Background

Annual influenza epidemics cause substantial morbidity and mortality and the 2009 H1N1 influenza outbreak was defined as a pandemic by the World Health Organisation (WHO). [1, 2]

About 50% of patients presenting to primary care with influenza-like illness (ILI) during a period of high influenza incidence, with some variation by country, usually have laboratory-confirmed influenza infection. [3] Unlike in Japan for example, [4] in European primary care, point-of-care testing for influenza is not routine practice, making it difficult for clinicians to distinguish influenza from other viral infections that cause ILI at the point of care with a high degree of certainty. [5] It is common practice throughout Europe to base treatment or advice to patients with ILI on signs and symptoms alone, given that point-of-care tests (POCTs) may neither be feasible nor perform adequately, and that antiviral treatment is hardly ever prescribed in primary care. [6] However, guidelines recommend use of antiviral drugs during the influenza season for high-risk groups. [7, 8] POCTs for routine use in primary care and (cost) effective treatment and willingness to prescribe would have major implications for care delivery. The performance of novel POCTs intended for use in primary care is seldom determined using samples obtained from patients consulting in primary care. [9] A valid, rapid test to detect influenza in primary care patients could help target antiviral treatment and informing patients about avoidance of risk for transmission and likely clinical course. Several new POCTS for common respiratory infections in primary care are merging onto the market. [10, 11] We aimed to use the opportunity of an existing trial, the “Antivirals for influenza-Like Illness? An rCt of Clinical and Cost effectiveness in primary CarE (ALIC^4^E)” trial, [12, 13] to evaluate the cobas® Liat® POCT, selected as it is easy to use and has excellent proof-of-principle supporting data. [14–18] However, before uptake into routine care, it is critical that analytic performance be established using samples obtained from real patients in the setting in which the test might be marketed.

Our primary objective was to assess the analytical performance of the cobas® Liat® POCT in detecting influenza A/B and RSV in samples collected from patients consulting with ILI in primary care. In addition, we aimed to explore ease of use.

## Methods

### Study endpoints

The primary endpoint was the sensitivity and specificity, and positive and negative predictive values of the cobas® Liat® POCT in comparison with laboratory-based PCR testing. The secondary endpoint was the acceptability and failure mode analysis of the cobas® Liat® POCT.

### Study population

We included a selection of swabs taken from patients presenting to primary care with symptoms of ILI, willing to participate in the ALIC^4^E trial, who met the inclusion criteria and gave informed consent. [12, 13] There were no additional exclusion criteria. For the purposes of the trial, ILI was defined as a sudden onset of self-reported fever, with at least one respiratory symptom (cough, sore throat, running or congested nose) and one systemic symptom (headache, muscle ache, sweats, chills or tiredness), with symptom duration of 72 h or less. [12, 13]

Recruitment for the ALIC^4^E trial was during periods of heightened influenza incidence which was determined by reviewing the data from the European Centre of Disease Prevention and Control (ECDC) in combination with local and regional alerts during the winters of 2015–2016, 2016–2017 and 2017–2018. The ALIC^4^E Trial team confirmed to each recruiting network when their sites could begin recruitment. Recruitment took place through recruiting sites (GP Practices, Out of Hours surgeries or Paediatric Centres); recruitment was paused when influenza activity dropped again below the epidemic threshold.

### Design

This was a method comparison study where a selection of swabs taken at baseline during the ALIC^4^E trial from participants presenting to primary care are analysed both on the cobas® Liat® POCT with the Influenza A/B & RSV Assay, as well as the laboratory-based Fast-Track Respiratory Pathogens 21 Plus kit (FTD, Fast-Track Diagnostics). The selection of swabs was aimed to detect a representative range of test positive and test negative samples for the viral pathogens relevant to the assay under investigation, including: all RSV positive samples, and, in function of the epidemiology of the circulating strains, a more or less equal proportion of samples testing positive for influenza A H1N1, influenza A H3N2 and influenza B.

For the laboratory-based PCR test, extraction was performed on NucliSENS easyMag (bioMérieux) and amplification on Lightcycler 480 (Roche).

Sample analysis on the cobas® Liat® POCT started 12th February 2018 and ended 30th June 2018.

For the POCT, all patients aged up to 16 years recruited during the ALIC^4^E trial had an oropharyngeal and nasal flocked swab taken at baseline by the responsible clinician or recruiter. These two samples were placed in one 3-ml Universal Transport Medium (UTM) (Copan). Those aged 16 years or older had a nasopharyngeal swab taken which was also placed in a 3-ml UTM. Once at the local laboratory, the samples were frozen and stored at − 70 °C (if a deep-freezer was not available on site at the local laboratory, storage at − 20 °C was deemed acceptable) and then transported to a central laboratory in Antwerp, Belgium. Samples went through a freeze/thaw cycle prior to aliquoting at the central laboratory in Antwerp.

A selection of the samples collected during the three influenza seasons (2015–2016, 2016–2017, 2017–2018) of the ALIC^4^E trial, frozen and stored at − 70 °C, were used, to ensure a sufficient large number of positive samples of the various viral pathogens. We enriched the sample for flu positivity.

The fresh samples collected in the third influenza season in Belgium were sent to the central laboratory for analysis both before and after a freeze-thaw cycle.

All laboratory analyses were done in the central laboratory at the University of Antwerp by trained lab technicians.

Prior to testing patient samples, the 2016 Quality Control for Molecular Diagnostics (QCMD) influenza panel (proficiency testing) was tested on the cobas® Liat® POCT, containing 10 samples with dilutions of different subtypes of influenza A and influenza B, to assess the performance of the POC device.

Samples with discordant results were analysed additionally by the RespiFinder 2Smart (PathoFinder).

The participant’s date of recruitment, gender and participant trial ID number were used as identifiers for these samples. Only the laboratory and trial team had access to this information for the purposes of sample identification and tracking.

### Acceptability and failure mode analysis

To assess acceptability and certain aspects of failure mode analysis, [19] the device operators were asked to complete a survey (see electronic supplementary material S1).

Acceptability was scored on a five-point Likert scale (1 = completely disagree to 5 = completely agree), including questions regarding device start-up, cartridge handling, operating the device, duration of the test, the provided manual and presentation of error codes. The risk of misinterpretation of failure modes was scored as: low risk–medium risk–high risk–difficult to assess, considering the following errors: expired cartridge, insufficient sample volume, incorrect cartridge insertion, unexpected test result and mismatch between the error code and actual error. The detection of the failure modes was scored as: always perceivable–probably perceivable–not perceivable for the same set of potential errors.

Operators were asked to list the strengths and weaknesses of the device.

### Sample size calculation and statistical analysis

We aimed to analyse a selection of swab results obtained using both platforms from a minimum of 730 participants recruited during three consecutive winters (samples selected from all stored frozen samples from season 1 and 2 and all new samples from season 3 until required sample size achieved), giving 90% power at 95% confidence to detect a maximum difference in results obtained from the two methods of 10% at a presumed sensitivity of 95%.

An expanded gold standard (EGS) approach was used to calculate sensitivities and specificities (positive by at least two tests). Results of this analysis were considered the reference standard. Sensitivity, specificity and positive and negative predictive value were determined. Wilson 95% confidence intervals were calculated with the Binom Package. All statistical analyses were performed using R 3.4.3, (https://www.r-project.org/).

## Results

### Quality control for molecular diagnostics panels

The 2016 influenza QCMD panel, containing 10 samples with dilutions of different subtypes of influenza A and B demonstrated excellent performance of the POC device (see electronic supplementary material S2.a).

### Description of population

Nasal and oropharyngeal swabs were obtained from 140 children and nasopharyngeal swabs from 604 adults (744 patients); median age was 33 years (range 1 to 88 years); 55.6% of patients were women, 91.8% had not been vaccinated against flu in the preceding 6 months and 93.3% had not received pneumococcal vaccination in the preceding 5 years (Table 1).

**Table 1:** Baseline characteristics of patients providing samples for this analysis.

		*N* = 744 (100%)
Age	Median (range), years	33 (1–88)
	12 months to < 5 years	37 (5.0%)
	5 years to < 18 years	103 (13.8%)
	18 years to < 65 years	560 (75.3%)
	+ 65 years	44 (5.9%)
Country of origin	Belgium	208 (28%)
	Poland	117 (15.7%)
	Spain	1 (0.1%)
	UK	99 (13.3%)
	Czech Republic	54 (7.3%)
	Denmark	17 (2.3%)
	France	13 (1.7%)
	Greece	21 (2.8%)
	Hungary	58 (7.8%)
	Ireland	9 (1.2%)
	Lithuania	72 (9.7%)
	The Netherlands	24 (3.2%)
	Norway	26 (3.5%)
	Sweden	25 (3.4%)
Sex	Male	330 (44.4%)
	Female	414 (55.6%)
Flu season	1 (2015–2016)	423 (56.9%)
	2 (2016–2017)	255 (34.3%)
	3 (2017–2018)	66 (8.9%)
Ethnicity	White	553 (74.3%)
	Black	3 (0.4%)
	Hispanic	4 (0.5%)
	Asian	6 (0.8%)
	Arabic	6 (0.8%)
	Other	23 (3.1%)
	Unknown	149 (20.0%)
Flu vaccination in the last 6 months	Yes	58 (7.8%)
	No	683 (91.8%)
	Unknown	3 (0.4%)
Pneumococcal vaccination in the last 5 years	Yes	37 (5.0%)
	No	694 (93.3%)
	Unknown	13 (1.7%)
Smoking status	Yes	121 (16.3%)
	No	591 (79.4%)
	Occasionally	32 (4.3%)
Sample type	Nasopharyngeal swab UTM	619 (83.2%)
	Oropharyngeal + nasal swab	125 (16.8%)
POC-PCR results	Negative rapid test	227 (30.5%)
	Positive rapid test for:	517 (69.5%)
	- influenza A	318 (42.7%)
	- influenza B	165 (22.2%)
	- RSV	30 (4.0%)

### Analytical performance

The analytical performance of the cobas® Liat® (Roche Diagnostics) POCT for diagnosing influenza A/B and RSV, and a laboratory-based platform, the Fast-Track Respiratory Pathogens 21 Plus kit (FTD, Fast-Track Diagnostics), was evaluated on 744 samples. Eleven samples with discordant results were analysed additionally by the RespiFinder 2Smart (PathoFinder). We encountered 8 run errors in total on the cobas® Liat®, resulting in 736 successfully run POCT tests.

Time to result for cobas® Liat® was within 22 min (2 min hands on time and 20 min analysis time).

The cobas® Liat® POCT had a sensitivity and specificity of 100% (95% CI 99–100%) and 98.1% (95%CI 96.3–99%) for influenza A, 100% (95% CI 97.7–100%) and 99.7% (95%CI 98.7–99.9%) for influenza B and 100% (95% CI 87.1–100%) and 99.4% (95%CI 98.6–99.8%) for RSV, respectively, with the EGS as reference standard (Table 2).

**Table 2:** Analytical performance of cobas® Liat® POCT on frozen samples using the Expanded Gold Standard (EGS) as reference

	TP	FP	FN	TN	Sensitivity (95% CI)	Specificity (95% CI)	PPV (95% CI)	NPV (95% CI)
cobas® Liat® influenza A	310	8	0	418	100% (99.0–100%)	98.1% (96.3–99.0%)	97.5% (95.1–98.7%)	100% (99.0–100%)
cobas® Liat® influenza B	163	2	0	571	100% (97.7–100%)	99.7% (98.7–99.9%)	98.8% (95.7–99.7%)	100% (99.3–100%)
cobas® Liat® RSV	26	4	0	706	100% (87.1–100%)	99.4% (98.6–99.8%)	86.7% (70.3–94.7%)	100% (99.5–100%)

*TP* true positives, *FP* false positives, *FN* false negatives, *TN* true negatives, *95% CI* 95% confidence intervals, *PPV* positive predictive value, *NPV* negative predictive value

The results for the fresh samples (*n* = 19) are shown in the electronic supplementary material S2.b, with similar findings and no samples with discordant results.

### Acceptability and failure mode analysis

**Fig. 1:**
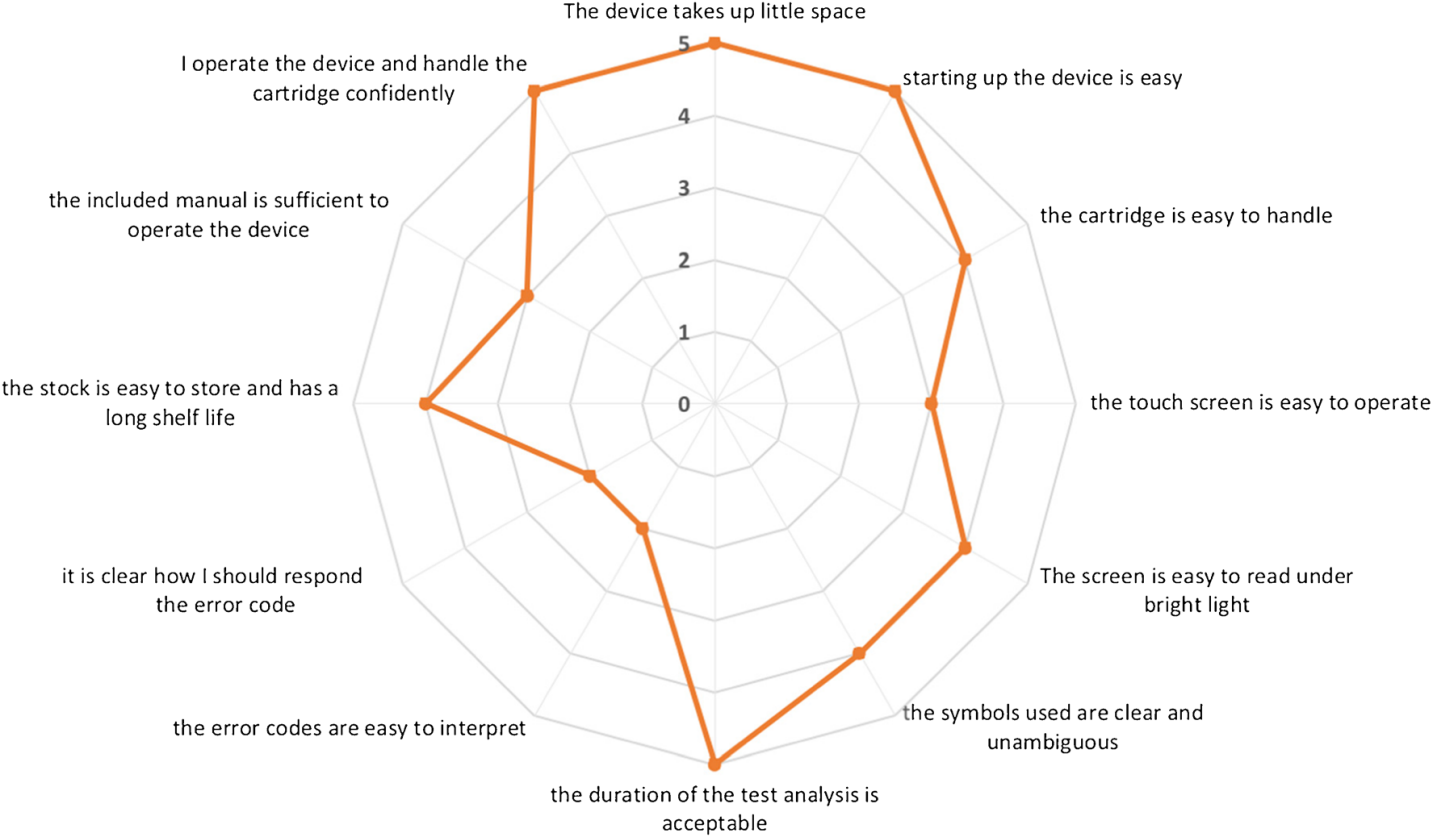
Acceptability of the POCT device

Five independent trained device operators (KJ, KL, KVDV, MK, VM) assessed the user-friendliness of the cobas® Liat® POCT device with a median acceptability of four out of five on the five-point Likert scale. Potential concerns identified were the touch screen readability and proficiency of the included manual (median scores of 3 out of 5) and interpretation of error codes and how to respond to them (median scores of 2 out of 5). (Fig. 1) The risk of misinterpretation of failure modes was generally considered low, apart from the risk of misinterpreting collection of an insufficient sample volume (deemed as high risk). Although most items regarding detection of failure modes were scored as ‘always perceivable’, agreement of an error code to the actual error was scored as ‘not perceivable’ by all raters.

The device’s size, analysis speed, ease of use and limited sample preparation were considered important strengths. The lack of a sample holder during pipetting, the size of the touch screen and difficulties reading the cycle threshold (Ct) values were generally considered potential weaknesses.

## Discussion

### Principal findings

The cobas® Liat® POCT demonstrated excellent analytical performance to detect influenza A, influenza B and RSV, and was considered easy to use, with a fast turn-around-time.

### Comparison with previous studies

The high sensitivity and specificity for influenza A, influenza B and RSV found in our study was comparable to previous findings, with better diagnostic accuracy than other routinely available point-of-care devices. [14–18, 20–30] The diagnostic accuracy of POCTs for influenza has been examined in individual studies and several systematic reviews. [31–33] Although the impact of the cobas® Liat® POCT on clinical decision making during visits to the emergency department has been established, [34] the effect on patient-relevant clinical outcomes still has to be determined.

### Strengths and limitations

We were able to collect paired samples in a real-life clinical setting of patients presenting to primary care with ILI in this adequately powered study, from 14 different countries across three influenza seasons. Samples were collected, transported and analysed in a standardised and rigorous way and compared to routinely available standard central laboratory multiplex tests. Sampling method varied by age which needs to be taken into account when considering the applicability of our results. Nasopharyngeal swabs were taken from adults and nasal and pharyngeal swabs were taken from children. Our results may therefore not be replicated fully through self-swabbing or nasal swabbing. While nasopharyngeal swabbing may provide the most accurate results, the choice of sample type for children was in accordance with routine care constraints, where more invasive methods are considered less appropriate.

Combining the samples from three different influenza seasons provided us with a representative range of influenza A and B samples, with very few cases of samples testing positive for RSV, which reflects clinical reality.

The vast majority of our samples were stored and frozen samples. However, fresh samples collected during season 3 indicated similar patterns, further strengthening our findings.

The cobas® Liat® POCT in our trial, was operated and maintained by trained lab technicians, familiar with standard operating procedures of POCT devices. This limits the generalisability of our findings to routine primary care both in terms of clinical performance and usability, even when the cobas® Liat® POCT would be performed by trained general practice staff. Although similar results were found in fresh samples, using frozen samples does not necessarily reflect routine primary care where samples would normally be analysed at the point of care, immediately following sampling.

### Implications for research

Studies of POCTs for influenza are needed, both in adults and children, with a particular evidence gap for primary care settings. Now that analytical performance has been established in primary care, trials of the impact on clinical course with cost-effectiveness analysis are required to establish the impact on patient-related outcomes, such as antibiotic prescribing rate, antiviral treatment and use of additional tests (chest radiography, blood cultures and routine blood tests). Combining influenza tests with other POCTs, such as C-reactive protein, may help rule out bacterial co-infection to reduce antibiotic prescribing rate in primary care settings.

### Implications for clinical practice

We have shown that the cobas® Liat® POCT is accurate, providing timely results in the hands of trained operators. However, the evidence to implement the test in primary care is still scarce. Considering the patient pathway is primordial to ensure adoption of a novel POCT. Time to result should ideally be less than 15 min to accommodate the already pressured consultation length in primary care. Furthermore, these tests should not substitute clinical appraisal. Clinicians should consider local initiatives before widespread implementation to evaluate costs, appropriateness and impact on further testing. The use of antiviral treatment and the impact of POCT to decide whether or not to prescribe still needs to be established and will guide future research on the role of POCT for influenza in primary care.

## Conclusions

Cobas® Liat® POCT is a promising decentralised test platform for detection of influenza A/B and RSV in primary care settings, provide fairly rapid results with excellent analytic performance.

## Electronic supplementary material

ESM 1 (PDF 115 kb)

ESM 2 (DOCX 19 kb)

## References

[1] Antonova EN, Rycroft CE, Ambrose CS, Heikkinen T, Principi N (2012) Burden of paediatric influenza in Western Europe: a systematic review. BMC Public Health 12. 10.1186/1471-2458-12-968 10.1186/1471-2458-12-968 PMC3534559 23146107

[2] World Health Organisation (2014) Influenza (seasonal); factsheet. WHO, Geneva

[3] Souty C, Masse S, Valette M, Behillil S, Bonmarin I, Pino C, Turbelin C, Capai L, Vilcu AM, Lina B, van der Werf S, Blanchon T, Falchi A, Hanslik T (2019) Baseline characteristics and clinical symptoms related to respiratory viruses identified among patients presenting with influenza-like illness in primary care. Clin Microbiol Infect 25(9):1147–1153 10.1016/j.cmi.2019.01.014 PMC7172742 30703528

[4] Maita H, Kobayashi T, Osawa H, Kato H (2018). Self-diagnosis of seasonal influenza in a rural primary care setting in Japan: a cross sectional observational study. PLoS One.

[5] Lee JJ, Verbakel JY, Goyder CR, Ananthakumar T, Tan PS, Turner PJ, Hayward G, Van den Bruel A (2019). The clinical utility of point-of-care tests for influenza in ambulatory care: a systematic review and meta-analysis. Clin Infect Dis.

[6] Adriaenssens N, Coenen S, Kroes AC, Versporten A, Vankerckhoven V, Muller A, Blix HS, Goossens H, Group EP (2011) European Surveillance of Antimicrobial Consumption (ESAC): systemic antiviral use in Europe. J Antimicrob Chemother 66 (8):1897–1905. doi:10.1093/jac/dkr190 10.1093/jac/dkr190 21622674

[7] Health NIf, Excellence C (2009) Amantadine, oseltamivir and zanamivir for the treatment of influenza (review of NICE Technology Appraisal Guidance 58). National Institute for Health and Clinical Excellence,

[8] Stephenson I, Clark TW, Pareek M (2008). Antiviral treatment and prevention of seasonal influenza: a comparative review of recommendations in the European Union. J Clin Virol.

[9] Verbakel JY, Turner PJ, Thompson MJ, Pluddemann A, Price CP, Shinkins B, Van den Bruel A (2017). Common evidence gaps in point-of-care diagnostic test evaluation: a review of horizon scan reports. BMJ Open.

[10] Verbakel JY, Aertgeerts B, Lemiengre M, Sutter AD, Bullens DM, Buntinx F (2014). Analytical accuracy and user-friendliness of the Afinion point-of-care CRP test. J Clin Pathol.

[11] Verbakel JY, Lemiengre MB, De Burghgraeve T, De Sutter A, Aertgeerts B, Shinkins B, Perera R, Mant D, Van den Bruel A, Buntinx F (2016). Should all acutely ill children in primary care be tested with point-of-care CRP: a cluster randomised trial. BMC Med.

[12] Bongard E, van der Velden AW, Cook J, Saville B, Beutels P, Munck Aabenhus R, Brugman C, Chlabicz S, Coenen S, Colliers A, Davies M, De Paor M, De Sutter A, Francis NA, Glinz D, Godycki-Cwirko M, Goossens H, Holmes J, Ieven M, de Jong M, Lindbaek M, Little P, Martinon-Torres F, Moragas A, Pauer J, Pfeiferova M, Radzeviciene-Jurgute R, Sundvall PD, Torres A, Touboul P, Varthalis D, Verheij T, Butler CC (2018). Antivirals for influenza-like illness? A randomised Controlled trial of Clinical and Cost effectiveness in primary CarE (ALIC(4) E): the ALIC(4) E protocol. BMJ Open.

[13] Butler CC, van der Velden AW, Bongard E, Saville BR, Holmes J, Coenen S, Cook J, Francis NA, Lewis RJ, Godycki-Cwirko M, Llor C, Chlabicz S, Lionis C, Seifert B, Sundvall P-D, Colliers A, Aabenhus R, Bjerrum L, Jonassen Harbin N, Lindbæk M, Glinz D, Bucher HC, Kovács B, Radzeviciene Jurgute R, Touboul Lundgren P, Little P, Murphy AW, De Sutter A, Openshaw P, de Jong MD, Connor JT, Matheeussen V, Ieven M, Goossens H, Verheij TJ (2019) Oseltamivir plus usual care versus usual care for influenza-like illness in primary care: an open-label, pragmatic, randomised controlled trial. Lancet. 10.1016/S0140-6736(19)32982-4

[14] Gibson J, Schechter-Perkins EM, Mitchell P, Mace S, Tian Y, Williams K, Luo R, Yen-Lieberman B (2017). Multi-center evaluation of the cobas((R)) Liat((R)) influenza A/B & RSV assay for rapid point of care diagnosis. J Clin Virol.

[15] Schmidt RLJ, Simon A, Popow-Kraupp T, Laggner A, Haslacher H, Fritzer-Szekeres M, Redlberger-Fritz M, Mayer FJ (2018) A novel PCR-based point-of-care method facilitates rapid, efficient, and sensitive diagnosis of influenza virus infection. Clin Microbiol Infect. 10.1016/j.cmi.2018.12.017 10.1016/j.cmi.2018.12.017 30583060

[16] Valentin T, Kieslinger P, Stelzl E, Santner BI, Groselj-Strele A, Kessler HH, Tiran B (2019). Prospective evaluation of three rapid molecular tests for seasonal influenza in patients presenting at an emergency unit. J Clin Virol.

[17] Akashi Y, Suzuki H, Ueda A, Hirose Y, Hayashi D, Imai H, Ishikawa H (2019) Analytical and clinical evaluation of a point-of-care molecular diagnostic system and its influenza A/B assay for rapid molecular detection of the influenza virus. J Infect Chemother. 10.1016/j.jiac.2019.02.022 10.1016/j.jiac.2019.02.022 30905631

[18] Maignan M, Viglino D, Hablot M, Termoz Masson N, Lebeugle A, Collomb Muret R, Mabiala Makele P, Guglielmetti V, Morand P, Lupo J, Forget V, Landelle C, Larrat S (2019). Diagnostic accuracy of a rapid RT-PCR assay for point-of-care detection of influenza A/B virus at emergency department admission: a prospective evaluation during the 2017/2018 influenza season. PLoS One.

[19] Chiozza ML, Ponzetti C (2009). FMEA: a model for reducing medical errors. Clin Chim Acta.

[20] Banerjee D, Kanwar N, Hassan F, Essmyer C, Selvarangan R (2018) Comparison of six sample-to-answer influenza A/B and respiratory syncytial virus nucleic acid amplification assays using respiratory specimens from children. J Clin Microbiol 56(11). 10.1128/JCM.00930-18 10.1128/JCM.00930-18 PMC6204686 30185508

[21] Gosert R, Naegele K, Hirsch HH (2019). Comparing the Cobas Liat influenza A/B and respiratory syncytial virus assay with multiplex nucleic acid testing. J Med Virol.

[22] Youngs J, Iqbal Y, Glass S, Riley P, Pope C, Planche T, Carrington D (2018) Implementation of the cobas Liat influenza point-of-care test into an emergency department during a high-incidence season: a retrospective evaluation following real-world implementation. J Hosp Infect. 10.1016/j.jhin.2018.12.008 10.1016/j.jhin.2018.12.008 PMC7124296 30562558

[23] Binnicker MJ, Espy MJ, Irish CL, Vetter EA (2015). Direct detection of influenza A and B viruses in less than 20 minutes using a commercially available rapid PCR assay. J Clin Microbiol.

[24] Chen L, Tian Y, Chen S, Liesenfeld O (2015). Performance of the Cobas((R)) influenza A/B assay for rapid Pcr-based detection of influenza compared to Prodesse ProFlu+ and viral culture. Eur J Microbiol Immunol (Bp).

[25] Nolte FS, Gauld L, Barrett SB (2016). Direct comparison of Alere i and cobas Liat influenza A and B tests for rapid detection of influenza virus infection. J Clin Microbiol.

[26] Melchers WJG, Kuijpers J, Sickler JJ, Rahamat-Langendoen J (2017). Lab-in-a-tube: real-time molecular point-of-care diagnostics for influenza A and B using the cobas(R) Liat(R) system. J Med Virol.

[27] Young S, Illescas P, Nicasio J, Sickler JJ (2017). Diagnostic accuracy of the real-time PCR cobas((R)) Liat((R)) influenza A/B assay and the Alere i influenza A&B NEAR isothermal nucleic acid amplification assay for the detection of influenza using adult nasopharyngeal specimens. J Clin Virol.

[28] Ling L, Kaplan SE, Lopez JC, Stiles J, Lu X, Tang YW (2018) Parallel validation of three molecular devices for simultaneous detection and identification of influenza A and B and respiratory syncytial viruses. J Clin Microbiol 56(3). 10.1128/JCM.01691-17 10.1128/JCM.01691-17 PMC5824037 29263204

[29] Goldstein EJ, Gunson RN (2019). In-house validation of the cobas Liat influenza A/B and RSV assay for use with gargles, sputa and endotracheal secretions. J Hosp Infect.

[30] Benirschke RC, McElvania E, Thomson RB Jr, Kaul KL, Das S (2019) Clinical impact of rapid point-of-care PCR influenza testing in an urgent care setting: a single-center study. J Clin Microbiol 57(3). 10.1128/JCM.01281-18 10.1128/JCM.01281-18 PMC6425177 30602445

[31] Koski RR, Klepser ME (2017) A systematic review of rapid diagnostic tests for influenza: considerations for the community pharmacist. J Am Pharm Assoc (2003) 57 (1):13–19. doi:10.1016/j.japh.2016.08.018 10.1016/j.japh.2016.08.018 27836481

[32] Merckx J, Wali R, Schiller I, Caya C, Gore GC, Chartrand C, Dendukuri N, Papenburg J (2017). Diagnostic accuracy of novel and traditional rapid tests for influenza infection compared with reverse transcriptase polymerase chain reaction: a systematic review and meta-analysis. Ann Intern Med.

[33] Petrozzino JJ, Smith C, Atkinson MJ (2010). Rapid diagnostic testing for seasonal influenza: an evidence-based review and comparison with unaided clinical diagnosis. J Emerg Med.

[34] Hansen GT, Moore J, Herding E, Gooch T, Hirigoyen D, Hanson K, Deike M (2018). Clinical decision making in the emergency department setting using rapid PCR: results of the CLADE study group. J Clin Virol.

