# Supplementary material for: Performance and ease of use of a molecular point-of-care test for influenza A/B and RSV in patients presenting to primary care
Source: Eur J Clin Microbiol Infect Dis. 2020 Mar 14;39(8):1453–60. doi: 10.1007/s10096-020-03860-5 (PMC7343728; doi:10.1007/s10096-020-03860-5)
Supplement: Supplementary file 1 — (PDF 115 kb) [file 10096_2020_3860_MOESM1_ESM.pdf]

Acceptability and Failure Mode Analysis: Roche Liat (ALIC4E)

*Required

1. **First Name and Surname** *

2. **Number of tests performed** *

3. **Acceptability** *

Mark only one oval per row.

	completely disagree	rather disagree	neutral	rather agree	completely agree
the device takes up little space					
the start-up of the device is easy					
the cartridges is easy to handle					
the touch screen is easy to operate					
the screen is easy to read under different lighting conditions					
the symbols used are clear and unambiguous					
the duration of the test analysis is acceptable					
the error codes are easy to interpret					
it is clear how I should respond to the error code					
the stock is easy to store and has a long shelf life					
the included manual is sufficient to operate the device					
I operate the device and handle the cartridge confidently					

4. **Risk of Misinterpretation** *

If one of these errors should occur, how big is the risk of misinterpretation?

Mark only one oval per row.

	low risk	medium risk	high risk	difficult to assess
the cartridge has expired or has already been used				
the sample volume is insufficient				
incorrect insertion of the cartridge into the device				
the result is unexpected or illogical				
the error code and the actual error do not match				

5. **Detection of failure modes** *

If one of these errors should occur, how likely is it that you would notice this error?

Mark only one oval per row.

	always perceivable	probably perceivable	not perceivable
the cartridge has expired or has already been used			
the sample volume is insufficient			
incorrect insertion of the cartridge into the device			
the result is unexpected or illogical			
the error code and the actual error do not match			

6. **Which are the strengths of this device according to you?**

7. **What are the shortcomings of this device?**

8. **Other remarks?**
