# Supplementary material for: Performance and ease of use of a molecular point-of-care test for influenza A/B and RSV in patients presenting to primary care
Source: Eur J Clin Microbiol Infect Dis. 2020 Mar 14;39(8):1453–60. doi: 10.1007/s10096-020-03860-5 (PMC7343728; doi:10.1007/s10096-020-03860-5)
Supplement: Supplementary file 2 — (DOCX 19 kb) [file 10096_2020_3860_MOESM2_ESM.docx]

**Electronic Supplementary Material S2.a:** a Quality Control for Molecular Diagnostics (QCMD) panel

| **Sample ID** | **QCMD expected result** | **cobas® Liat**® **result instrument 1** | **cobas® Liat**® **result instrument 2** |
| --- | --- | --- | --- |
| InfRNA16-01 | INFB (Yamagata) | Not tested | Positive for INFB |
| InfRNA16-02 | INFA (H3N2) | positive for INFA | Not tested |
| InfRNA16-03 | INFB (Yamagata) | Not tested | positive for INFB |
| InfRNA16-04 | negative for INFA & negative for INF B | negative for INFA & negative for INF B | negative for INFA & negative for INF B |
| InfRNA16-05 | INFA (H5N1) | Not tested | positive for INFA |
| InfRNA16-06 | INFA (H3N2) | positive for INFA | Not tested |
| InfRNA16-07 | INFA (H1N1) | positive for INFA | Not tested |
| InfRNA16-08 | INFB (Victoria) | Not tested | positive for INFB |
| InfRNA16-09 | INFA (H1N1) | positive for INFA | Not tested |
| InfRNA16-010 | INFB (Victoria) | Not tested | positive for INFB |

**INFA: Influenza A; INFB: Influenza B**

**Electronic Supplementary Material S2.b:** analytical performance of cobas® Liat® POCT on fresh samples using the Expanded Gold Standard (EGS) as reference

|  | **TP** | **FP** | **FN** | **TN** | **Sensitivity**  **(95% CI)** | **Specificity**  **(95% CI)** | **PPV**  **(95% CI)** | **NPV**  **(95% CI)** |
| --- | --- | --- | --- | --- | --- | --- | --- | --- |
| **cobas® Liat**® **Influenza A** | 7 | 0 | 0 | 12 | 100%  (64.6-100%) | 100%  (75.8-100%) | 100%  (64.6-100%) | 100%  (75.8-100%) |
| **cobas® Liat**® **Influenza B** | 7 | 0 | 0 | 12 | 100%  (64.6-100%) | 100%  (75.8-100%) | 100%  (64.6-100%) | 100%  (75.8-100%) |
| **cobas® Liat**® **RSV** | 0 | 0 | 0 | 19 | All samples (n=19) tested negative for RSV | | | |

**TP: true positives, FP: false positives, FN: false negatives, TN: true negatives; 95% CI: 95% confidence intervals, PPV: positive predictive value, NPV: negative predictive value**
